# Low Noise Feed-Through Compensation Circuit Design for Resonant MEMS Pressure Sensor

**DOI:** 10.3390/mi16040400

**Published:** 2025-03-29

**Authors:** Jialuo Liao, Pinghua Li, Jiaqi Miao, Ruimei Liang, Zhongfeng Gao, Xuye Zhuang

**Affiliations:** College of Mechanical Engineering, Shandong University of Technology, Zibo 255000, China; 15550345181@163.com (J.L.); lipinghua@sdut.edu.cn (P.L.); 13581071927@163.com (J.M.); lrm25790@163.com (R.L.); gzf15550340109@163.com (Z.G.)

**Keywords:** resonant pressure sensor, charge amplifier, noise optimization, feed-through effect

## Abstract

The feed-through effect of resonant pressure sensors usually introduces interfering noise signals, leading to the degradation of sensitivity, linearity, and other performances of the sensor test system. A low-noise charge amplifier and its feed-through compensation circuit are designed to realize high-precision measurements. The designed improved charge amplifier has a differential common-source structure as the output buffer stage, which can effectively reduce the output noise of the circuit while increasing the input impedance, thus improving the accuracy of the feed-through compensation coefficient. By establishing the equivalent circuit model of the sensor and analyzing the influence of the feed-through effect on the sensor test, the feed-through compensation circuit is designed to suppress the feed-through signal. Experimental testing of the sensor proves that the designed circuit can effectively suppress the feed-through effect of the sensor. The noise power spectral density of the improved charge amplifier is tested to be 26.74 nV/√Hz, which is a 65% reduction in noise density. The feed-through compensation circuit eliminates the interference frequency of 34,919 Hz introduced by the feed-through capacitor. Additionally, the resonance peak of the intrinsic resonance frequency of the pressure sensor is −40.75 dBV, which is reduced by 8 dBV compared with that before the feed-through compensation. The feed-through compensation circuit effectively reduces the feed-through interference signal of the sensor, improves the measurement accuracy of the test system, and provides technical support for the design of a low-noise, high-precision, stable, and reliable sensor test system.

## 1. Introduction

Silicon micro-resonant pressure sensors with miniaturization, low power consumption, high precision, good stability, and other advantages in the civil direction, industrial automation aerospace, and other fields have been widely used. At the same time, these pressure sensors are more complex in realizing pressure conversion and are more affected by external vibration, requiring vibration isolation packages. These challenges also place higher demands on the measurement accuracy, stability, and sensitivity of the sensor detection circuit. As a key factor affecting the performance of resonant sensors, the study of sensor test systems is mainly focused on reducing circuit noise and improving measurement accuracy [1].

The weak capacitance detection circuit, as the front-end interface circuit of a resonant pressure transducer, is one of the main noise sources of the test system. Existing weak capacitance signal detection techniques are mainly categorized into trans-impedance amplifiers and charge amplifiers. Trans-impedance amplifiers can provide high gain as well as low noise levels, but the stability of the circuit deteriorates further at high gain, resulting in lower resolution and sensitivity; although the low noise performance of charge amplifiers is not as good as that of trans-impedance amplifiers, such circuits are characterized by high resolution and high sensitivity, and provide better signal isolation and anti-interference capability, ensuring the stability and accuracy of the circuit measurement that is suitable for precision measurements. Wang et al. developed a low-noise photodetector based on a single JFET and charge amplifier to optimize the input voltage noise through the ultra-low noise performance of the JFET. The circuit has a maximum signal-to-noise ratio of 15 dB, which is suitable for the detection of weak signals [2]. Zeng et al. proposed a charge amplifier based on a bootstrap common source and common gate structure. The circuit uses a single-ended input to optimize the frequency characteristics of the circuit while ensuring low noise performance; its charge sensitivity is 33 V/pC, and the maximum signal-to-noise ratio is 35:1 [3]. Robert Oven added a unit gain buffer between the output of the charge amplifier circuit and the feedback resistor to reduce the effect of stray capacitance on the feedback capacitance and to reduce the measurement error [4]. Emad Alnasser et al. proposed a charge amplifier using a single op-amp loop instead of a feedback resistor. The circuit has an output noise RMS of 94 μV and an equivalent input charge of 18.8 fCrms while achieving a low cutoff frequency and low noise [5]. Zhang et al. effectively reduced the input common-mode voltage drift by using input common-mode feedback and modulation–demodulation techniques. The sensitivity and dynamic range of the circuit are 12.53 V/pF and 102 dB, respectively, to further improve the detection accuracy of weak capacitance [6].

In addition, in order to enhance the frequency signal output from the resonant pressure sensor and suppress the common-mode noise, its detection circuit generally uses the traditional three-op-amp differential charge amplification structure, which requires a high degree of matching of numerous op-amps and electronic components in order to achieve the desired common-mode suppression effect, and the output noise of the circuit will be increased exponentially, which in turn will affect the signal detection of the sensor [7]. Simple single op-amp differential charge amplifier circuits have poor common-mode rejection, although they can reduce the matching requirements of electronic components and circuit output noise. To address these issues, Massarotto et al. reported a differential charge amplification circuit based on a two-stage topology. The function of the first stage of this circuit is to convert the differential mode charge into a single-ended signal while suppressing the common-mode noise; the second stage adopts a standard charge amplification structure. Although the circuit structure is capable of achieving high common-mode rejection, it is actually a deformation of the trans-impedance amplifier, giving up the high input impedance advantage of the charge amplifier [8,9]. Thus, to enhance the detection circuit’s noise performance, the differential charge amplifier’s circuit structure offers scope for further refinement.

Silicon micro-resonant pressure sensors fabricated from SOI (Silicon-On-Insulator) chips typically operate in electrostatic drive/capacitance detection mode. The substrate material is semiconductor silicon. On this basis, a parasitic capacitance, also known as feed-through capacitance, exists between the metal electrode on the sensor chip and the substrate. Consequently, part of the sensor’s drive signal directly transmits to the detection electrode via the feed-through capacitance, a phenomenon known as the feed-through effect of the sensor [10]. The feed-through effect causes the sensor’s detection signal to contain part of the drive interference signal, constituting another significant noise source in sensors. Consequently, in practical pressure sensor applications, it is essential to suppress this feed-through effect as much as possible. Ma et al. employed Electromechanical Amplitude Modulation (EAM) to eliminate the feed-through effect on the piezoresistive cantilever microprobe. This technique utilizes a high-frequency carrier signal to modulate the detection signal to a high frequency and achieves separation of the feed-through signal from the detection signal through modulation/demodulation, thereby improving signal quality [11]. Wu et al. proposed a feed-through canceling circuit based on an inverting amplifier. This method suppresses the feed-through effect by adjusting the compensation circuit’s output voltage, generating a compensation voltage signal equal in amplitude but opposite in phase to the feed-through signal [12]. Owing to the complexity and uncertainty of sensor processing technology, the test circuit for suppressing the feed-through effect requires ongoing structural improvements to enhance measurement accuracy in practical applications.

A modified charge amplifier featuring a differential common-source structure as the input buffer stage is designed to reduce the circuit output noise in a resonant pressure transducer test system. The circuit effectively suppresses input common-mode noise signals, with its low-noise performance enhancing the accuracy of the feed-through compensation factor. Analysis via an equivalent circuit model reveals that the feed-through effect introduces interference frequencies in the sensor’s frequency response curve. The proposed feed-through compensation circuit, based on a modified charge amplifier, is capable of effectively suppressing the sensor’s feed-through effect. The detection circuit incorporating feed-through compensation eliminates the interference frequency caused by the feed-through capacitor. It also eliminates the effect of the feed-through signal on the resonant peak value of the pressure sensor. Consequently, the measurement accuracy of the pressure sensor test system is improved.

## 2. Basic Principles and Design of Charge Amplifier

### 2.1. Basic Principles of Charge Amplifier

**Figure 1:**
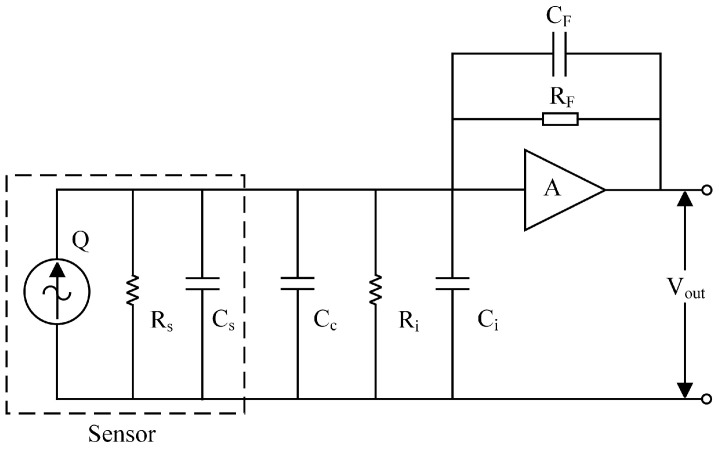
The basic structure of a charge amplifier.

A charge amplifier circuit primarily functions as a current integrator. It converts weak signals into voltage signals via current integration and transforms a high-impedance charge source (e.g., a resonant pressure transducer) into a low-impedance voltage source, thereby minimizing test signal loss and ensuring high-fidelity measurements. Figure 1 illustrates a typical equivalent circuit structure of a resonant pressure sensor and charge amplifier [13].

Where *Q* denotes the sensor’s output charge signal; *R_S_* represents the sensor’s equivalent resistance; *C_S_*, the sensor’s equivalent capacitance; *C_C_*, the input stray capacitance; *R_i_* and *C_i_*, the op-amp’s input resistance and capacitance; and *R_F_* and *C_F_*, the op-amp’s feedback resistance and capacitance. When the op-amp’s open-loop gain *A* is sufficiently large (typically 10⁴ to 10⁶), the following relationship can be derived:

(1) $$\left(1+A\right)/R_{F}\gg 1/R_{s}+1/R_{i}$$

(2) $$\left(1+A\right)C_{F}\gg C_{s}+C_{i}+C_{c}$$

When the sensor’s equivalent capacitance *C_s_*, equivalent resistance *Rs*, input stray capacitance *C_c_*, and the op-amp’s input resistance *R_i_* and input capacitance *C_i_* are negligible in the charge amplifier circuit, the output voltage *V_out_* of the charge amplifier can be expressed as follows:

(3) $$V_{\mathrm{out}}=\frac{-sAQ}{\left(\frac{1}{R_{F}}+sC_{F}\right)\left(1+A\right)}$$

When the detected signal’s frequency satisfies 1/*R_F_* ≪ *ωC_F_*, the term 1/*R_F_* becomes negligible, allowing the above equation to be simplified as follows:

(4) $$V_{\mathrm{out}}=\frac{-sAQ}{s\left(1+A\right)C_{F}}=-\frac{AQ}{\left(1+A\right)C_{F}}\approx -\frac{Q}{C_{F}}$$

Analysis shows that the output voltage *V_out_* of the circuit is independent of the size of the parasitic capacitance, which can effectively suppress the influence of the parasitic capacitance. The output charge *Q* of the sensor and the feedback capacitance *C_F_* affect the voltage *V_out_*, which indicates that the feedback capacitance *C_F_* determines the circuit sensitivity of the charge amplifier. Therefore, the fidelity of the detected signal can be significantly improved by using the charge amplifier to capture the weak charge signal of the pressure sensor.

### 2.2. Design of Charge Amplifier

**Figure 2:**
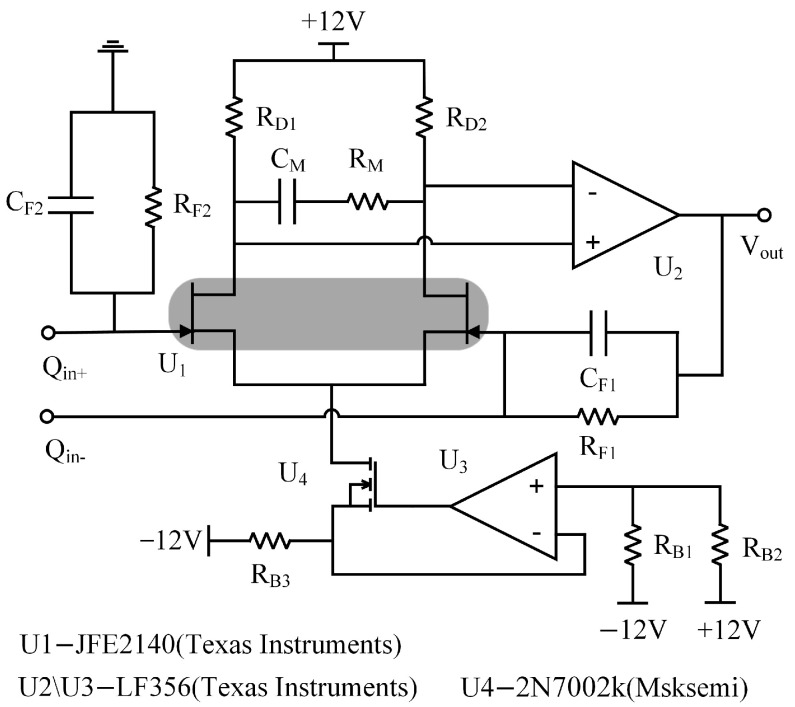
Diagram of the improved differential charge amplifier.

Conventional three-op-amp differential charge amplifier circuits involve a large number of electronic components and require high circuit matching. While these conventional differential charge amplifiers enhance common-mode rejection, they do so at the expense of degraded noise performance. To tackle these challenges, the proposed improved differential charge amplifier, featuring a fully differential common-source structure as the input buffer stage, effectively overcomes the limitations of conventional differential charge amplifier circuits. It offers superior common-mode rejection and optimized noise performance for differential charge amplifiers. Figure 2 illustrates the structure of the improved differential charge amplifier, which employs a fully differential common-source structure as the input buffer stage. The overall differential charge amplifier circuit comprises an input buffer stage, a constant current source, and an operational amplifier stage.

When the resistive-capacitive feedback networks *R_F_*_1_, *C_F_*_1_, and the resistive-capacitive canceling networks *R_F_*_2_, *C_F_*_2_ satisfy *R_F_*_1_ = *R_F_*_2_ = *R_F_* and *C_F_*_1_ = *C_F_*_2_ = *C_F_* [14], according to Equation (3), the differential-mode output voltage, *V_DM_*, of this improved differential charge amplifier can be expressed as:

(5) $$V_{DM}=Q_{DM}\left(\frac{1}{C_{F1}}+\frac{1}{C_{F2}}\right)=\frac{2Q_{DM}}{C_{F}}$$

The common-mode noise at the differential input is passed through the modified differential charge amplifier, and the output common-mode voltage *V_CM_* is given by the following equation:

(6) $$V_{CM}=Q_{CM}\left(\frac{1}{C_{F1}}-\frac{1}{C_{F2}}\right)=0$$

According to Equation (5), the overall charge gain *G* of the improved differential charge amplifier is given by the following equation:

(7) $$G=\frac{V_{DM}}{Q_{DM}}=\frac{2}{C_{F}}$$

(1) Input buffer stage

To enhance the charge amplifier’s signal-to-noise ratio and minimize circuit input noise, a high-input-impedance buffer stage can be added before the op-amp. The high-performance JFE2140 differential pair from Texas Instruments (Suzhou Branch, Suzhou, Jiangsu Province, China) features ultra-low noise, low input capacitance, ultra-high transconductance, and extremely high input impedance. The charge amplifier’s input buffer stage employs a fully differential common-source structure, offering high input impedance. This significantly reduces the impact of source impedance mismatch on common-mode rejection ratio (CMRR) performance and enables differential input. The input buffer stage comprises a JFE2140 common-source differential pair (U_1_) loaded with resistors R_D1_ and R_D2_.

(2) Constant Current Source Bias Circuit

A JFET and op-amp-based constant-current source provides a stable bias current, enabling the input buffer stage to operate in the saturation region. When the JFE2140’s drain-source current (*I_DS_*) is 5 mA, it delivers ultra-low noise performance with extremely high input impedance, producing an input reference noise of 0.9 nV/√Hz and a forward transconductance of 20 mS. This ensures that the input buffer stage meets low-noise charge amplifier requirements. The input buffer stage’s fully differential common-source structure results in a total leakage current of 10 mA through the JFE2140. In this design, U_4_ utilizes the low-noise MOSFET 2N7002K (Msksemi Semiconductor, Hsinchu, Taiwan Province, China), while U_3_ employs the low-noise, high-speed LF356 operational amplifier to form the constant-current source circuit. When powered by a ±12 V supply, set R_B1_ = 20 kΩ, R_B2_ = 10 kΩ, and R_B3_ = 1 kΩ. Based on the junction field-effect transistor’s transfer characteristics [15]:

(8) $$I_{D}=I_{DSS}{\left(1-\frac{U_{GS}}{U_{GS(off)}}\right)}^{2}$$

where *I_D_* is the drain current, *I_DSS_* is the saturated drain current, *U_GS_* is the gate source voltage, and *U_GS_* (*off*) is the pinch-off voltage.

At this time, *I_D_* = 10.3 mA can be obtained. The magnitude of the drain current is approximately equal to the magnitude of the source current (*I_D_* ≈ *I_S_*). The constant-current source circuit provides a leakage current of *I_DS_* ≈ 10 mA. At this time, the single-sided field-effect transistor’s input buffer stage experiences a leakage current of approximately 5 mA, enabling U_1_ to operate at its optimal performance.

(3) Operational Amplifier Stage

Differential input charge amplifiers typically amplify differential signals while suppressing common-mode interference, thereby enhancing detected signal noise performance. The operational amplifier stage employs Texas Instruments’ low-noise, high-precision LF356, featuring low noise and a high gain-bandwidth product, making it suitable for the improved charge amplifier’s amplifier stage. As shown in Figure 2, differential pairs of common-source structures U_1_ receive differential signal input and connect differentially to operational amplifier stage U_2_. This single-op-amp differential charge amplifier circuit not only enhances interference immunity but also exhibits lower noise and power consumption compared to other differential circuits. The input differential charge signal passes through the resistive-capacitive feedback network at the inverted input and the resistive-capacitive offset network at the in-phase input, respectively, to achieve the amplification of the differential signals and to cancel out the interference of the common-mode charge signals. After comprehensive consideration, the improved differential charge amplifier’s feedback resistance is set to 100 MΩ, and the feedback capacitance to 100 pF.

## 3. Simulation and Testing of Circuit Performance

To validate the common-mode rejection performance and noise level of the designed improved differential charge amplifier circuit, comprehensive simulations, analyses, and experimental tests are conducted.

### 3.1. Simulation of Common-Mode Rejection Capability

**Figure 3:**
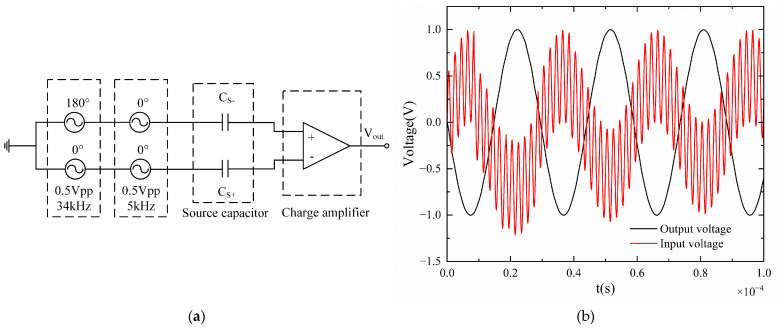
Simulation of common-mode rejection capability: (**a**) Circuit simulation model; (**b**) results of time–domain simulation.

To simulate the circuit’s common-mode rejection capability, an analog charge source model is established for the weak charge signals output by the resonant pressure sensor. The charge source comprises a voltage source and a front-end capacitor (C_S+_ = C_S−_). The voltage source exhibits an amplitude of 0.5 V_pp_ and a frequency of 34 kHz. The series capacitance is 100 pF, while the common-mode interference signal has an amplitude of 0.5 V_pp_ and a frequency of 5 kHz. The simulated circuit model is presented in Figure 3a. The time–domain waveform curve of the circuit simulation is shown in Figure 3b. Simulation results indicate that the improved differential charge amplifier circuit successfully realized the amplification of differential charge signals and effectively suppressed input common-mode interference signals.

### 3.2. Testing of the Noise Power Spectrum

**Figure 4:**
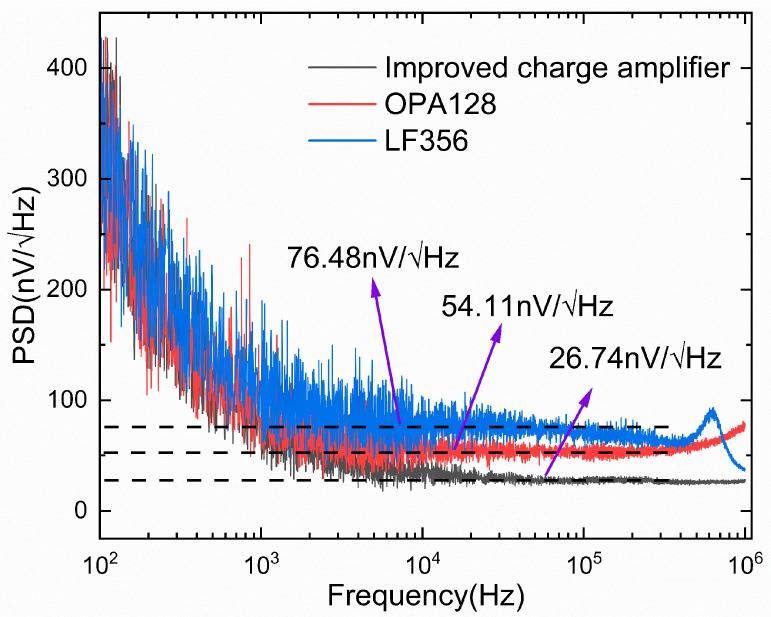
Measurement of noise power spectral density.

Circuit noise power spectral density (PSD) characterizes the distribution of noise power across different frequencies in a circuit. It indicates the circuit’s noise level, aiding in noise source identification and characteristic analysis. Noise test experiments for noise power spectral density scanning were conducted on the traditional three-op-amp differential charge amplifiers (LF356, OPA128–Texas Instruments Semiconductor Technology (Suzhou) Co., Ltd. Suzhou, Jiangsu Province, China) and the improved differential charge amplifier (LF356–Texas Instruments Semiconductor Technology (Suzhou) Co., Ltd. Suzhou, Jiangsu Province, China), as depicted in Figure 4. Figure 4 reveals that conventional three-op-amp differential charge amplifiers exhibit substantial noise power. The high-precision electrostatic-grade OPA128 exhibits a voltage noise density of 54.11 nV/√Hz, while the standard-precision LF356 shows 76.48 nV/√Hz. In contrast, the proposed improved differential charge amplifier circuit achieves a voltage noise density of 26.74 nV/√Hz, representing respective noise density reductions of 50.6% and 65% compared to the aforementioned two differential charge amplifiers. Compared to the conventional three-operational-amplifier charge amplifier, the improved differential charge amplifier demonstrates a substantial enhancement in noise performance. In subsequent sensor feed-through compensation experiments, employing low-noise charge amplifiers notably enhances the accuracy of feed-through compensation coefficients. This effectively mitigates feed-through effect interference in sensor testing, further improving sensor performance.

## 4. Analysis of the Feed-Through Effect

### 4.1. Principle of the Feed-Through Effect

**Figure 5:**
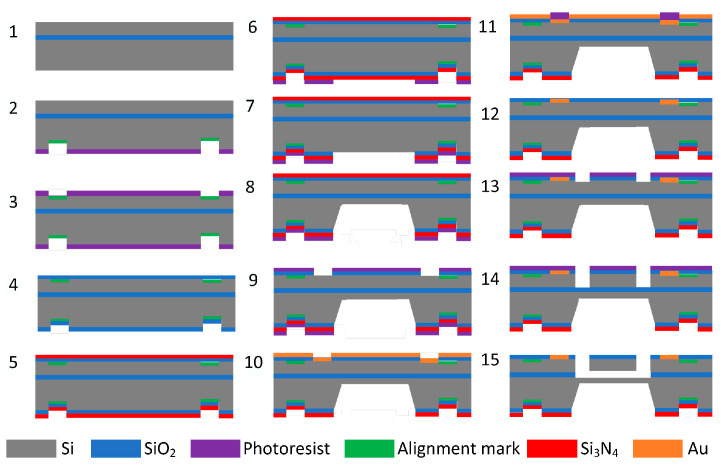
Fabrication process of the resonant pressure sensor.

In this study, SOI (N-type <100>) wafers were used to fabricate pressure sensors. Initially, SOI wafers (top silicon 35 μm, buried oxygen layer 3 μm thick, 30,000 Å ± 500 Å, bottom silicon 400 μm thick) were prepared as shown in Figure 5(1). This was followed by backside lithography and front-side lithography and thermal oxygen growth of SiO_2_, as shown in Figure 5(2–4). LPCVD deposited Si_3_N_4_ with a thickness of 1000 Å as a blocking layer for KOH etching as shown in Figure 5(5–8), and backside lithography was performed, thereby opening the etching window for KOH solution. Si_3_N_4_ was etched using RIE with an etch thickness of 1000 Å, BOE etched SiO_2_ with a thickness of 3000 Å, and KOH solution (concentration: 30%, temperature 80 °C) etched the back cavity to an etch depth of 325 μm. As shown in Figure 5(9), photolithography was carried out to lithograph the metal dot window, remove the Si_3_N_4_, and etch the oxide layer. The next step is to sputter Au on the upper surface with a thickness of 200 nm, perform photolithography, and etch the Au layer to make the metal dots, as shown in Figure 5(10–12). Subsequently, photolithography is performed to make the resonance structure graphical and etch the structure of the resonance layer, as shown in Figure 5(13,14). Finally, scribing is performed to release the chip structure and complete the device processing, as shown in Figure 5(15).

**Figure 6:**
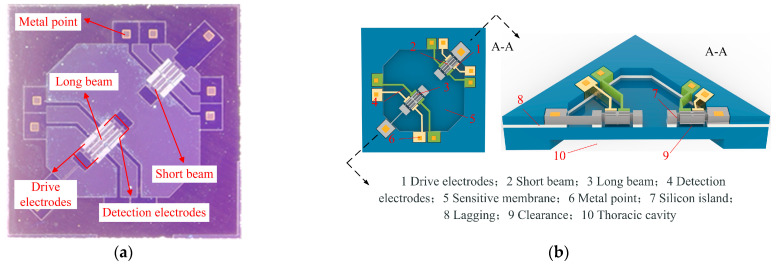
Schematic representation of resonant pressure sensor: (**a**) The top-view photograph of the device; (**b**) schematic representation of the device structure.

Figure 6 presents the top view and structural diagram of the resonant pressure sensor. The studied resonant pressure sensor features a dual resonator structure, comprising a long beam and a short beam. Positioned at the middle and edge of the pressure-sensitive diaphragm, they maximize the pressure-frequency response. The device enhances resonator sensitivity through differential output. Each resonator in the sensor adopts a double-beam resonator structure, generating symmetrical modes. This configuration suppresses nonlinear vibration, enhances device stability and vibration uniformity, and improves the coupling effect.

**Figure 7:**
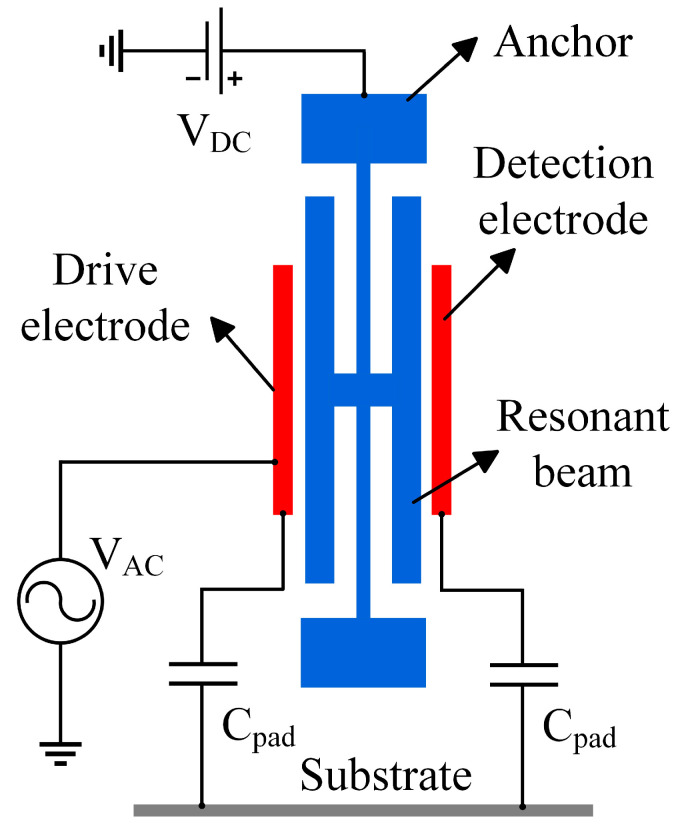
Schematic representation of the feed-through effect.

Figure 7 shows the schematic diagram of the feed-through effect of the resonant pressure sensor. Due to the presence of feed-through capacitance C_pad_ in the silicon micro-resonant pressure sensor chip itself, the interference resulting from the coupling of the drive signal with the detection signal is called the feed-through effect. Under the influence of the feed-through capacitance, part of the sensor’s drive signal is transmitted directly to the detection circuit, which is called the feed-through signal. The feed-through signal is not generated by the actual vibration of the pressure sensor; the signal will make the sensor output frequency signal not accurately reflect the measured pressure changes. The feed signal and pressure changes caused by the change of the detection signal law are different, resulting in the relationship between the pressure sensor output frequency and the pressure value producing a nonlinear error, affecting the measurement accuracy. If the measured pressure value change is slight, the pressure sensor output frequency change is also slight; the feed signal may mask the frequency change, reducing the signal-to-noise ratio of the detection signal. Pressure sensors require greater pressure changes to produce detectable frequency changes, reducing the sensitivity of the pressure sensor. All these effects lead to serious deviations of the measured resonant frequency and quality factor from the actual values, which cannot truly reflect the performance of the device and, in severe cases, can lead to the inability of the entire pressure test system to test the input external parameters. Due to the complex structure and precision processing of resonant pressure sensors, it is difficult to eliminate the feed-through effect from the structure. Therefore, this paper considers the use of circuit compensation technology to suppress the feed-through effect of the sensor.

**Figure 8:**
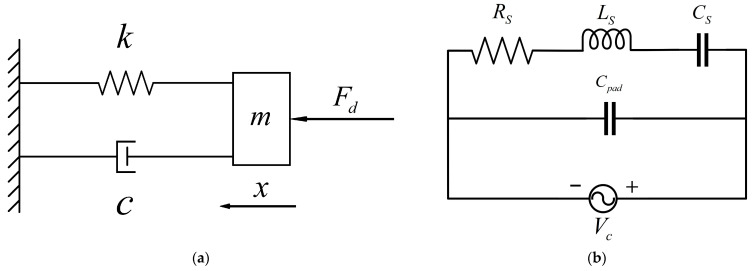
Modeling of short beam resonator: (**a**) Second-order damping system; (**b**) equivalent electrical model.

Preliminary tests revealed a significant feed-through effect in the short beam resonator of the resonant pressure sensor. In a pressure-free vacuum environment, its initial resonant frequency is 34,964 Hz. When the resonant sensor works, its mechanical parameters and electrical parameters can be transformed into each other to establish the corresponding equivalent electrical model. Consequently, an equivalent circuit model for the short beam resonator can be developed to analyze the influence of the feed-through effect on the pressure sensor test. When the short-beam resonator is in the resonant state, the resonator structure can be equated to a mass-spring-damped second-order system, as shown in Figure 8a. Where *m* is the equivalent mass of the resonator, *k* is the equivalent stiffness of the resonator, *c* is the equivalent damping coefficient of the resonator, *F_d_* is the electrostatic driving force applied to the resonator, and *x* is the small displacement of the resonator. The equivalent electrical model of the short beam resonator is established based on the equivalent mechanical model of the resonant sensor [16], as shown in Figure 8b. Where *R_S_* is the equivalent resistance of the short beam resonator, *L_S_* is the equivalent inductance, *C_S_* is the equivalent capacitance, and *C_pad_* is the equivalent feed-through capacitance of the short beam resonator.

According to Figure 8a, the kinetic equation of the resonator can be obtained from Newton’s second law, and the equation is denoted as:

(9) $$m\ddot{x}(t)+c\dot{x}(t)+kx(t)=F_{d}=A_{d}sin(\omega_{d}t)$$

where *A_d_* is the amplitude of the driving force and *ω_d_* is the angular frequency of the driving force. Equation (9) can be simplified to:

(10) $$\ddot{x}(t)+\frac{\omega_{n}}{Q}\dot{x}(t)+\omega_{n}^{2}x(t)=\frac{A_{d}\sin\left(\omega_{d}t\right)}{m}$$

where *ω_n_* denotes the intrinsic angular frequency of the resonator and *Q* denotes the quality factor of the resonator. The sensor’s drive voltage is formed by superimposing DC and AC components. This configuration determines the driving force magnitude, as shown in the following equation:

(11) $$F_{d}=-\frac{dE}{dx}=-\frac{\varepsilon_{0}\varepsilon_{r}S}{2{\left(x_{0}+x\right)}^{2}}{\left[V_{AC}\sin(\omega_{d}t)+V_{DC}\right]}^{2}$$

where E is the energy carried by the capacitor, *S* is the relative area of the capacitor, vacuum permittivity *ε*_0_ = 8.85418 × 10^−12^ F/m, *ε_r_* is the relative permittivity of the capacitor, and *x*_0_ is the initial distance between the two plates of the capacitor. It can be seen that the electrostatic force is a nonlinear function of the driving voltage and vibration displacement. Typically, in resonant sensors, the static gap *x*_0_ between the resonant beam and metal electrodes is significantly larger than the vibration displacement *x*. Then, Equation (11) can be localized by performing a Taylor expansion at *x*_0_ to achieve local linearization, which can be simplified to:

(12) $$F_{d}\approx -\frac{\varepsilon_{0}\varepsilon_{r}S}{2x_{0}^{2}}{\left[V_{AC}\sin(\omega_{d}t)+V_{DC}\right]}^{2}+\frac{\varepsilon_{0}\varepsilon_{r}S}{x_{0}^{3}}x{\left[V_{AC}\sin(\omega_{d}t)+V_{DC}\right]}^{2}$$

In the equation, the first term represents the electrostatic attraction between the two plates of the capacitor, which is usually used to drive the resonant beam and is independent of the displacement change *x* of the resonant beam. The second term represents the repulsive force, which is linearly related to the displacement variation *x* of the resonant beam and affects the stiffness factor of the resonant beam. Substituting Equation (12) into Equation (9) reduces to:

(13) $$\begin{array}{l} m\ddot{x}(t)+c\dot{x}(t)+\left\{k-\frac{\varepsilon_{0}\varepsilon_{r}S}{x_{0}^{3}}{\left[V_{AC}\sin(\omega_{d}t)+V_{DC}\right]}^{2}\right\}x(t)\\ =-\frac{\varepsilon_{0}\varepsilon_{r}S}{2x_{0}^{2}}{\left[V_{AC}\sin(\omega_{d}t)+V_{DC}\right]}^{2} \end{array}$$

Neglecting the effect of electrostatic force on the stiffness of the resonant transducer, the above equation can be simplified to:

(14) $$m\ddot{x}(t)+c\dot{x}(t)+kx(t)=-\frac{\varepsilon_{0}\varepsilon_{r}S}{2x_{0}^{2}}{\left[V_{AC}\sin(\omega_{d}t)+V_{DC}\right]}^{2}$$

The sensor employs differential technology and single-frequency electrostatic drive, permitting neglect of the DC and octave terms in Equation (14). The equation can be simplified to:

(15) $$m\ddot{x}(t)+c\dot{x}(t)+kx(t)=\frac{2C_{0}V_{AC}V_{DC}\sin(\omega_{d}t)}{x_{0}}$$

Equation (16) is obtained by derivation:

(16) $$m\ddot{v}(t)+c\dot{v}(t)+kv(t)=\frac{2C_{0}V_{DC}}{x_{0}}V_{AC}cos(\omega_{d}t)$$

where *v* is the speed of vibration of the resonator. Furthermore, during sensor operation, a weak capacitive signal emerges between the resonator and differential detection electrodes. Under the DC bias voltage, the charge signal undergoes displacement, generating a weak current output, which can be represented as [16]:

(17) $$i(t)=2\frac{dQ}{dt}=2V_{DC}\frac{dC}{dt}=2V_{DC}\frac{dC}{dx}\frac{dx}{dt}=\frac{2C_{0}V_{DC}}{x_{0}}\frac{dx}{dt}$$

When K = 2*C*_0_*V_DC_*/*x*_0_, the above equation can be simplified to:

(18) $$i(t)=K\frac{dx}{dt}=Kv(t)$$

According to Figure 8b, the second-order differential equation of this equivalent electrical model can be expressed as:

(19) $$L\ddot{i}(t)+R\dot{i}(t)+\frac{1}{C}i(t)=\frac{V_{AC}\sin(\omega t)}{dt}=V_{AC}cos(\omega t)$$

Substituting Equation (18) into Equation (19), the above equation reduces to:

(20) $$KL\ddot{v}(t)+KR\dot{v}(t)+\frac{K}{C}v(t)=V_{AC}cos(\omega t)$$

In addition:

(21) $$\left\{\begin{array}{l} \omega =\sqrt{k/m_{0}}\\ \alpha =c/2m_{0}\omega \\ Q=1/\left(2\alpha \right)=\sqrt{km_{0}}/c \end{array}\right.$$

where *ω* is the intrinsic angular frequency, *α* is the damping ratio, and *Q* is the quality factor. Comparing Equations (16) and (20), it is obtained that:

(22) $$\left\{\begin{array}{l} L=\frac{m}{K^{2}}\;\\ R=\frac{c}{K^{2}}=\frac{m\omega }{K^{2}Q}\\ C=\frac{K^{2}}{k}=\frac{1}{\omega^{2}L} \end{array}\right.$$

The series resonant frequency of the equivalent electrical model is obtained at this point:

(23) $$f=\frac{1}{2\pi \sqrt{LC}}$$

The specific parameters of the equivalent electrical model are calculated based on the above equation, and the specific parameters of the equivalent electrical model are shown in Table 1.

**Figure 9:**
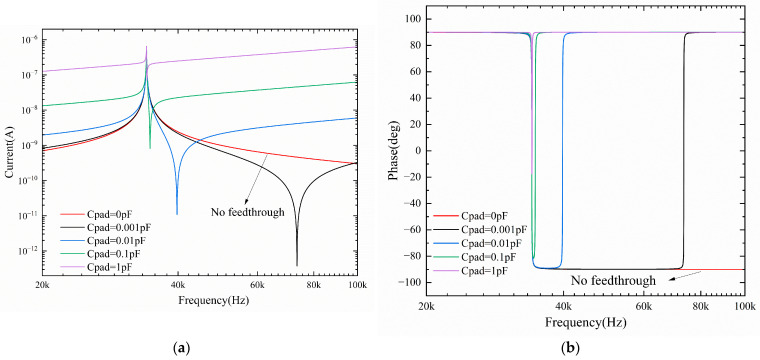
Simulation results of feed-through effect: (**a**) Amplitude–frequency characteristic curves; (**b**) phase–frequency characteristic curves.

When the equivalent circuit model of the short beam resonator exists with different sizes of feed-through capacitance, the influence of the feed-through effect on its frequency response curve is shown in Figure 9. From the simulation results, it can be seen that when there is a feed-through effect in the equivalent circuit model of the sensor, two resonant frequencies appear in its frequency characteristic curve. This is manifested in the amplitude–frequency characteristic curve by the appearance of two resonance peaks, one with a very large value and one with a very small value. The extreme value of the resonance peak for the resonance frequency of the sensor 34 kHz, the very small value of the resonance peak for the equivalent circuit of the feed-through capacitor introduced by the interference frequency. The phase–frequency characteristic curve of the equivalent circuit shows a sudden phase change corresponding to the resonance peak. When the feed-through capacitance of the sensor is increased, the amplitude–frequency characteristic curve of the equivalent circuit is shifted upward, the resonance frequency of the maximum value is shifted, and the resonance peak of the minimum value is gradually close to the resonance peak of the maximum value. When the feed-through capacitance is large enough, the resonance peaks corresponding to the extreme values on the amplitude–frequency characteristic curve coincide with the resonance peaks corresponding to the very small values, and there is no sudden phase change on the phase–frequency characteristic curve. This is specifically shown by the absence of resonant peaks, resonant frequencies, and corresponding phase transitions on the resonator’s sweep curve.

The above analysis indicates that the feed-through effect not only introduces interference frequencies into the sensor’s frequency response curve but also causes the sensor’s output signal to contain numerous feed-through signals. Severe feed-through signals can entirely overwhelm the sensor’s output useful signals, leading to the entire sensor test system’s failure.

### 4.2. Feed-Through Compensation Circuit

For the feed-through effect of silicon micro-resonant pressure sensors, differential detection techniques, EAM techniques, feed-through compensation circuits, and feed-through compensation algorithms are often used to realize the reduction or elimination of feed-through signals [17,18,19,20]. In addition, resonant pressure sensors using a piezoelectric drive/piezoresistive mode of operation can reduce the feed-through effect of the pressure sensor. However, it requires the use of special piezoelectric materials or strain gauges, etc. The choice of materials is relatively limited, and the manufacturing process may be more complex, resulting in higher costs. Due to the aging of the material, temperature changes, and other factors, its output signal may drift, resulting in reduced accuracy and stability. Sensors using electrostatic actuation/capacitive detection technology, on the other hand, have lower power consumption and higher accuracy and stability. This type of pressure sensor usually uses materials such as monocrystalline silicon to make the pressure membrane and sensitive elements, and the choice of materials is more flexible. With the development of microelectromechanical systems (MEMS) technology, its manufacturing cost is gradually reduced, and it can realize mass production. Therefore, it is of practical application to study the feed-through effect generated by resonant pressure sensors under the electrostatic actuation/capacitive detection technique.

The differential detection circuit’s ability to suppress feed-through signals is predicated on the device exhibiting good symmetry. Owing to manufacturing errors in device processing technology, process technology level, structural symmetry, and other factors, the symmetry of the MEMS resonant device falls short of the ideal state or is even suboptimal. In practice, while the resonant sensor test system can utilize a differential detection circuit to suppress the device’s feed-through effect, it cannot fully eliminate this effect. Consequently, differential drive and detection technology is employed to suppress the feed-through effect at its generation end. Additionally, a feed-through compensation circuit is designed to completely eliminate the feed-through effect.

**Figure 10:**
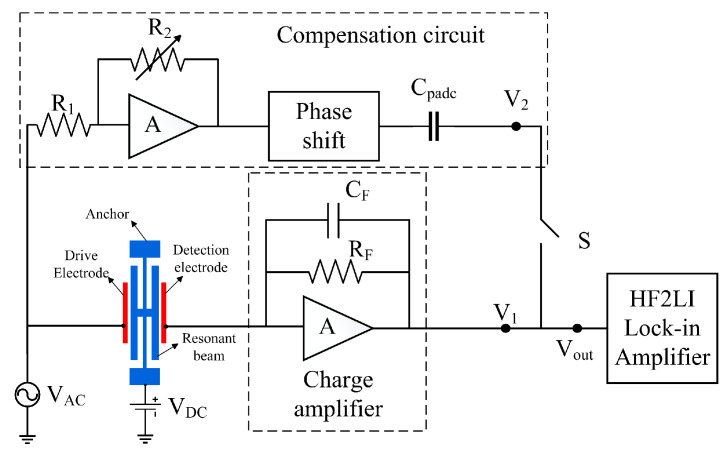
Diagram of the feed-through compensation circuit.

The feed-through compensation circuit is based on the sensor test circuit, an additional compensation circuit to suppress the feed-through effect. It does not depend on the symmetry of the device itself, nor does it need to reduce the electrostatic drive efficiency. Theoretically, it can eliminate the feed-through effect and apply it to resonant pressure sensors with varying performance parameters. Figure 10 illustrates the equivalent circuit of the feed-through compensation circuit. No DC bias voltage is applied to the resonator, and the AC drive voltage is in the mV range. This implies the resonator is not resonated, with the feed-through effect generating a feed-through signal at the detection electrode. The AC drive voltage on the drive electrode splits into two paths: one passes through the device’s feed-through capacitor, generating a feed-through signal that the charge amplifier outputs as AC voltage V_1_; the other travels through the amplifier and phase-shift circuits, which control amplitude and phase via compensating capacitor C_padc_ to produce voltage V_2_. V_2_ matches V_1_ in amplitude but is opposite in phase, counteracting the feed-through voltage and thus nullifying the sensor’s feed-through effect. The above analysis shows that the feed-through compensation accuracy of the resonant pressure sensor mainly depends on the noise performance of the charge amplifier, amplitude control of the amplifier circuit, and phase shift circuit’s phase control.

### 4.3. Compensated Experimental Testing

The feed-through compensation experiment tests the sensor using a feed-through compensation circuit based on differential drive and differential detection, with the compensation coefficient test scheme depicted in Figure 10. The laboratory-prepared resonant pressure sensor serves as the test object, with the sensor test system established as per Figure 10. The test equipment comprises a HF2LI lock-in amplifier (Zurich Instruments AG, Technoparkstrasse, Zurich, Switzerland), a DC power supply, a vacuum chamber, and a test circuit. The HF2LI lock-in amplifier, featuring an oscilloscope and frequency sweep function, supplies an AC drive voltage V_AC_. The DC power supply provides a DC bias voltage V_DC_ for the transducer, while the vacuum chamber maintains a 2 Pa vacuum level to minimize air damping.

Disconnect switch S and use the oscilloscope function to record the circuit noise of the detection circuit noted as noise N_1_. The signal generator outputs an AC drive signal with an amplitude of 200 mV and a phase of 0° to act on the sensor. Since the sensor chip is not applied to the DC bias voltage, the device is in a quiescent state; at this time, the detection circuit output signals are feeder signals. After determining the feed-through signal, open the switch S of the feed-through compensation circuit and adjust the size and phase of the compensation voltage V_2_. The compensated circuit output noise N_2_ is comparable to the original circuit noise N_1_ of the circuit, thus suppressing the feed-through effect of the resonant pressure sensor. At this time, the DC bias voltage is applied to the sensor, the device resonates, and the signals detected by the detection circuit are all resonant signals of the device’s vibration. Post-testing, the feed-through compensation circuit’s compensation coefficients are determined as amplification coefficient A = 0.568 and phase shift angle φ = −127°.

**Figure 11:**
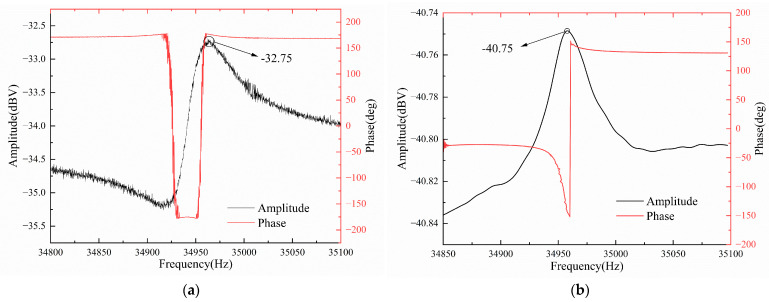
Sweep curves of resonant pressure sensor: (**a**) Sweep curves with uncompensated circuit; (**b**) sweep curves with compensated circuit.

With the DC bias voltage V_DC_ set at 20 V and the AC drive voltage V_AC_ at 200 mV for the resonant pressure sensor. Figure 10 illustrates the experimental results comparing the sensor’s frequency sweep with and without the feed-through compensation circuit. The black curve indicates the amplitude variation during the frequency sweep test, while the red curve shows the phase variation. As shown in Figure 11a, when there is no feed-through compensation circuit, two resonant peaks appear on the amplitude–frequency characteristic curve of the pressure sensor. The resonance frequency corresponding to the very small value is the interference frequency introduced by the feed-through capacitor, 34,919 Hz, and the resonance frequency corresponding to the very large value is the intrinsic resonance frequency of the sensor, 34,964 Hz, with a resonance peak of −32.75 dBV. This resonance peak includes the effect of feed-through signals, which, in practical applications of sensors, can lead to a decrease in the sensitivity and linearity of the pressure sensor. Also, the phase–frequency characteristic curve undergoes a sudden phase shift near these two resonant frequencies.

As shown in Figure 11b, when the feed-through compensation circuit is connected, the interference frequency of 34,919 Hz introduced by the feed-through capacitor and its resonance peak disappear on the sweep curve, and the corresponding phase mutation also disappears. This shows that the feed-through compensation circuit can realize feed-through compensation or even completely eliminate the feed-through effect of the sensor to improve the detection quality. After the realization of feed-through compensation, the resonance peak of the pressure sensor is −40.75 dBV, and the resonance peak is reduced by 8 dBV, which significantly reduces the impact of the feed-through signal on the resonance peak. At this time, the resonant frequency of the pressure sensor is measured to be 34,958 Hz, and the quality factor Q is 1326. The experimental test results are consistent with the simulation analysis results of the equivalent circuit model, which further illustrates the correctness of the equivalent electrical model of the sensor.

By comparing with most of the current feed-through effect suppression techniques [10,18,19,20,21,22,23,24], the resonant pressure sensor used in this paper has a slightly lower quality factor Q, but the proposed feed-through compensation circuit can also achieve higher compensation accuracy. Compared with Zhang et al. who used a half-frequency driving technique to excite a resonator with low Q, the feed-through compensation circuit de-signed in this paper can effectively suppress the feed-through effect of the resonant pressure sensor without decreasing the driving efficiency, eliminating the interference frequency of the pressure sensor, and reducing the resonance peak by 60%.

## 5. Conclusions

To mitigate the resonant pressure sensor test system’s interference noise, this paper presents a low-noise charge amplifier and its feed-through compensation circuit, both based on a differential common-source structure. With the charge amplifier’s differential common-source structure serving as an input buffer stage, it effectively decreases circuit output noise, thereby enhancing the feed-through compensation coefficient’s accuracy. Using the sensor’s equivalent circuit model, analysis shows that the feed-through effect introduces interference frequencies on the sensor’s frequency response curve. The designed feed-through compensation circuit effectively suppresses this interference during sensor testing. Experimental results indicate the improved charge amplifier’s noise power spectral density is 26.74 nV/√Hz, marking a 65% reduction in noise density. The feed-through compensation circuit eliminates the 34,919 Hz interference frequency caused by the feed-through capacitor. At this point, the pressure sensor’s intrinsic resonance frequency resonance peak is −40.75 dBV, representing an 8 dBV reduction. This significantly diminishes the feed-through signal’s impact on the resonance peak. The designed feed-through compensation circuit enhances the test system’s measurement accuracy and offers valuable insights for sensor test system research. The feed-through compensation circuit proposed for resonant pressure sensors can also be applied to suppress the feed-through effect of other MEMS sensors, thus improving the measurement accuracy of the sensor.

## Figures and Tables

**Table 1:** Parameters of the equivalent circuit model.

Inductance L_S_ (H)	Resistance R_S_ (Ω)	Capacitance C_S_ (fF)	Equivalent Mass m (kg)	Equivalent Stiffness k (N/m)	Equivalent Capacitance c (F)
5987	63,951	3.65	5.1844 × 10^−10^	23.66	5.8853 × 10^−14^

## Data Availability

The original contributions presented in the study are included in the article; further inquiries can be directed to the corresponding author.

## References

[1] Lv M.C., Li P.H., Miao J.Q., Qiao Q., Liang R.M., Li G.L., Zhuang X.Y. (2024). Design and Optimization of MEMS Resonant Pressure Sensors with Wide Range and High Sensitivity Based on BP and NSGA-II. Micromachines.

[2] Wang J., Wu S.e., Mi C., Qiu Y., Bai X.a. (2023). A low-noise, high-gain, and large-dynamic-range photodetector based on a JFET and a charge amplifier. Front. Inf. Technol. Electron. Eng..

[3] Zeng G., Hu C., Wei S., Yang J., Li Q., Ge L., Tan C. (2017). Research and development of a high-performance differential-hybrid charge sensitive preamplifier. Appl. Radiat. Isot..

[4] Oven R. (2014). Modified Charge Amplifier for Stray Immune Capacitance Measurements. IEEE Trans. Instrum. Meas..

[5] Dobrev D.P., Alnasser E., Neycheva T.D. (2021). Lossy Integrator Readout Circuit With Active Bias Point. IEEE Sens. J..

[6] Zhang X., Li P., Zhuang X., Sheng Y., Liu J., Gao Z., Yu Z. (2023). Weak Capacitance Detection Circuit of Micro-Hemispherical Gyroscope Based on Common-Mode Feedback Fusion Modulation and Demodulation. Micromachines.

[7] Yantao S., Ning X., Wejinya U.C., Li W.J. High sensitivity 2-D force sensor for assembly of surface MEMS devices. Proceedings of the 2004 IEEE/RSJ International Conference on Intelligent Robots and Systems (IROS) (IEEE Cat. No.04CH37566).

[8] Massarotto M., Carlosena A., Lopez-Martin A.J. (2008). Two-Stage Differential Charge and Transresistance Amplifiers. IEEE Trans. Instrum. Meas..

[9] Giannelli P., Calabrese G., Frattini G., Granato M., Capineri L. (2019). A Buffered Single-Supply Charge Amplifier for High-Impedance Piezoelectric Sensors. IEEE Trans. Instrum. Meas..

[10] Zhang M.-N., Wang R., Dong L., Wang L.-F., Huang Q.-A. (2024). Analysis and Mitigation of the Feedthrough Capacitance in Weakly Coupled Silicon Resonators. IEEE Sens. J..

[11] Ma T., Fahrbach M., Peiner E. (2025). Piezoresistive Cantilever Microprobe with Integrated Actuator for Contact Resonance Imaging. Sensors.

[12] Wu Y., Fan C., Gu L., Liu M., Wu X., Cui F. (2024). Feedthrough effect in MEMS gyroscopes and fully differential feedthrough cancellation method. Rev. Sci. Instrum..

[13] Wang C., Li Y., Jia L., Zhang S., Ye J. (2020). Design of charge-sensitive and current-sensitive preamplifiers for electrostatic sensor. J. Electrostat..

[14] Yamashita M. (2002). Amplification Circuit for Electric Charge Type Sensor. U.S. Patent.

[15] Razavi B. (2005). Design of Analog CMOS Integrated Circuits.

[16] Mayberry C.L. (2014). Interface Circuits for Readout and Control of a Micro-Hemispherical Resonating Gyroscope. Master’s Thesis.

[17] Teng G., Yang C., Quan A., Li C., Li H., Cheng Y., Chang H., Kraft M., Zhang H. (2025). Extracting mechanical quality factor and eliminating feedthrough using harmonics of thermal-piezoresistive micromechanical resonators. Microsyst. Nanoeng..

[18] Fan C., Wu Y., Gu L., Wang Z., Liu W., Cui F. (2023). Automatic feedthrough cancellation methods for MEMS gyroscopes. Proc. Inst. Mech. Eng. Part C J. Mech. Eng. Sci..

[19] Wang Z., Zhang W., Gu L., Liu M. (2021). Feedthrough cancellation for MEMS gyroscope using the calculation method via digital circuit. Proceedings of the International Conference on Autonomous Unmanned Systems.

[20] Wang Y., Zheng H., Hu Y., Sun X., Duan J. (2024). Electrical-feed-through elimination in micro-hemisphere resonator measurement based on mixed-frequency-excitation of different harmonic signals. Sens. Actuators A.

[21] Chen D., Zhang H., Sun J., Pandit M., Sobreviela G., Wang Y., Zhang Q., Seshia A., Xie J. Phase-controlled oscillation in a capacitive nonlinear ring resonator with on-chip feedthrough de-embedding. Proceedings of the 2020 IEEE 33rd International Conference on Micro Electro Mechanical Systems (MEMS).

[22] Zhang Y., Bao J.-F., Li X.-Y., Zhou X., Wu Z.-H., Zhang X.-S. (2020). Fully-Differential TPoS Resonators Based on Dual Interdigital Electrodes for Feedthrough Suppression. Micromachines.

[23] Wang Y., Liu Y., Xu H., Li Z., Li Z. (2023). A Wideband and Low Reference Spur PLL with Clock Feedthrough Suppressed and Low Current Mismatch Charge Pump and Symmetrical CML Divider. Electronics.

[24] Setiono A., Nelfyenny, Nyang’au W.O., Peiner E. (2024). Replicating Spectral Baseline for Unambiguous Frequency Locking in Resonant Sensors. Sensors.

